# Engaging, interacting and connecting with our learners and teachers

**DOI:** 10.1007/s40037-013-0092-x

**Published:** 2013-10-30

**Authors:** A. D. C. Jaarsma

**Affiliations:** Evidence-Based Education, Amsterdam Medical Centre, University of Amsterdam (AMC-UvA), PO Box 22660, 1100 DD Amsterdam, the Netherlands

In the first editorial of this year’s issue of *Perspectives on Medical Education,* Jan Borleffs [1] wrote about medical education being and becoming future proof: ‘Definitely, educational concepts based on previous approaches with proven effectiveness should be preserved, but the content of the programme needs to change in concurrence with the changes in society and the requirements for good health care in the next decades’.

In this issue of PME, Jamiu Busari opens our eyes towards these changes in society and what the challenges are for good and sustainable health care, by ‘using’ the discourse of generation segmentation. Busari focuses on the changing needs and expectations of different generations as consumers of health care and as learners in the medical education domain. He characterises the new Millennial physician (born: 1985–2005) as ‘an expert in the science of medicine and (the interrelationships between) the social sciences and humanities related to clinical care’ [2].

This is enforced by a letter to this journal from two students from the USA. I presume both Millennial learners, make a plea for more attention to the humanities in medicine and medical education Ramai and Goldin [3], Their arguments for truly connecting and interacting with patients are very much in line with how Busari describes the needs of Millennial health consumers.

Jerardi et al. [4] describe Millennial learners in their Show and Tell paper. They share with us how learners were more engaged during morning report educational sessions by using technology and entertainment to increase interaction. The authors were slightly disappointed that the intervention group (i.e. using multimedia, audience participation and faculty mentorship) did not show better results on knowledge retention testing. This is on the one hand understandable because, as Kirkpatrick [5] stated earlier (1996), engagement and fun seem to be essential conditions for learning. On the other hand, the attempts to engage students may also distract from learning: in my daily dialogues with teachers and educators, I often hear the ‘complaint’ about the new generation of learners: ‘that everything should be fun and (visual) entertainment and that students’ attention is difficult to hold without it.’ From this viewpoint, one might argue that it is good to see that there was no decline in knowledge despite engaging learners more. Empirical studies with better outcome measures on knowledge retention and deep understanding are necessary to show us how this may work.

I think this issue of PME, with the papers introduced above, shows that the generation of learners of today and tomorrow needs to feel connected, interacted, and engaged and will then work hard and have fun! A helpful paper in this context may be the recently published ‘Twelve tips for facilitating Millennials’ learning’ in Medical Teacher [6].

The factors affecting engagement and motivation of *teachers* in an academic hospital setting were studied by Van den Berg et al. In their valuable paper, they found that feedback on teaching performance is one of the strongest predictors for teaching engagement. The teachers’ age, and therefore their belongingness to a certain ‘generation group’, did not seem to influence their results [7]. When comparing these results to the overview given by Busari, there seems to be a discrepancy. Busari says that: ‘Baby Boomers (born: 1945–1964) may feel insulted by feedback’. However, ‘Gen X’ers (born: 1965–1984) feel more at home with feedback and are less/not dependent on immediate and continuous feedback’.

Understanding the complex nature of different generations’ relationships to medical education, in my opinion, calls for developing a more thorough theoretical background. More empirical studies are needed to support the idea of groups of different generations. It may well be that differences are not so much related to certain age groups as more to individuals.

Dekker et al. at least show in their empirical study that, at an individual level, inappropriate behaviours in student–teacher encounters are perceived in a variety of ways. They found differences between groups of students and teachers on written vignettes describing misconduct and (sexual) harassment, but no clear pattern emerged. The authors propose, as a practical implication for medical education, that teachers and students discuss vignettes on unprofessional behaviour together, with the purpose of creating more awareness of professional boundaries in relationships and differences in perceptions [8]. This suggestion does fall into place with how Millennial learners (if we can still refer to them as such) prefer to learn: in dialogue and connection with peers and teachers [2] while being able to express their fears, hopes, and stress in a secure arena [3].

This issue—written by and for our next generation of health care professionals—contributes to the ongoing dialogue of making our domain become more and more future proof. I sincerely hope you enjoy the readings and please join in whenever you feel your ideas, innovations and research contribute to our dialogues. PME strives to engage, interact and connect with you, our readers and authors, continuously.
